# Supplementary material for: CDK12 promotes tumorigenesis but induces vulnerability to therapies inhibiting folate one-carbon metabolism in breast cancer
Source: Nat Commun. 2022 May 12;13:2642. doi: 10.1038/s41467-022-30375-8 (PMC9098894; doi:10.1038/s41467-022-30375-8)
Supplement: Supplementary file 1 — Supplementary Information [file 41467_2022_30375_MOESM1_ESM.pdf]

## SUPPLEMENTARY INFORMATION

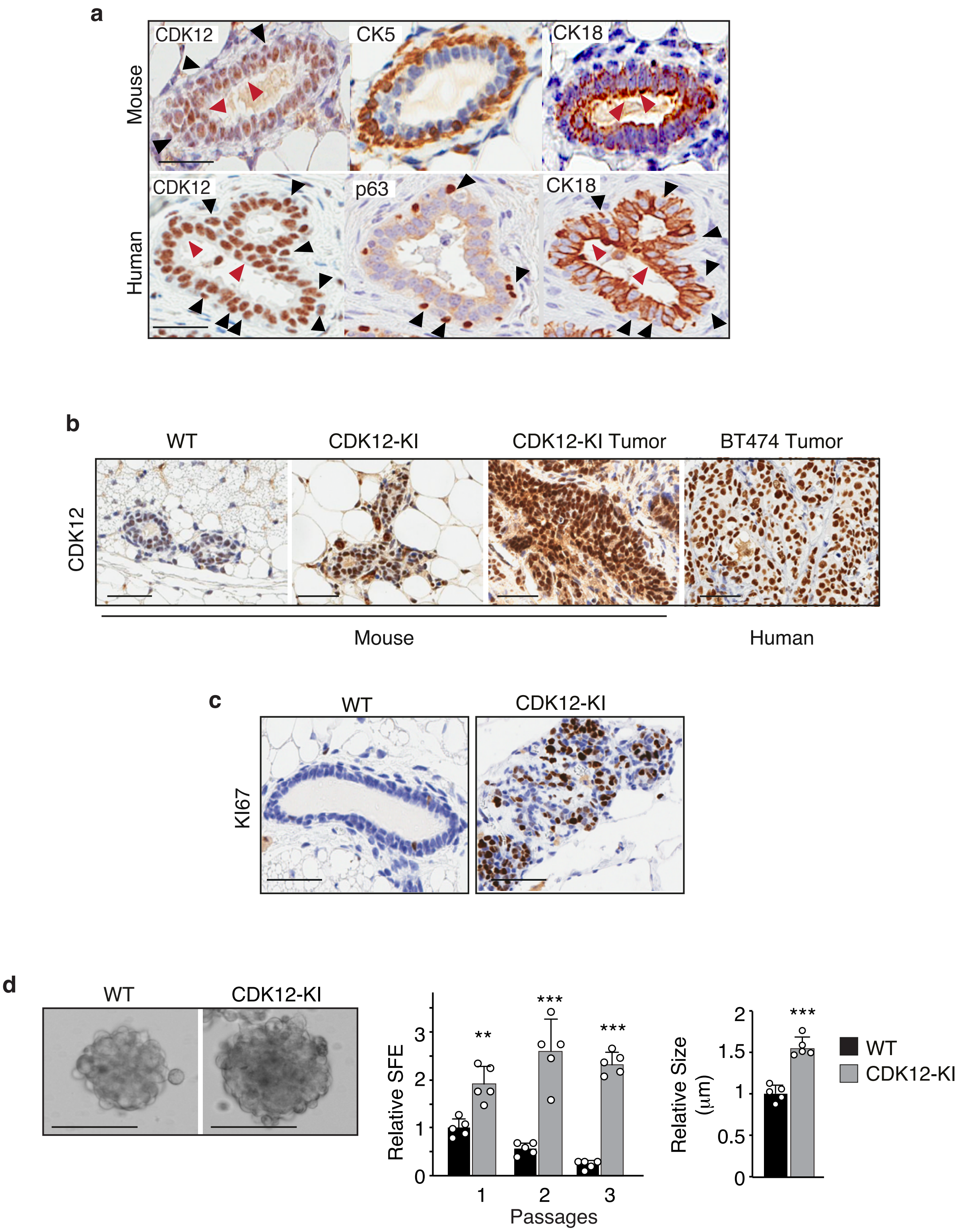


Supplementary Figure 1

**Supplementary Figure 1. CDK12 overexpression enhances spontaneous and carcinogen-induced breast tumor formation in vivo and the appearance of transformed phenotypes in vitro.** **a.** Expression pattern of endogenous CDK12 in WT FVB mouse (top panels) and human (bottom panels) mammary glands, determined by IHC analysis of FFPE sections. Cytokeratin (CK)-5 and p63 are markers for the basal/myoepithelial layer in mouse and human, respectively. CK18 identifies the luminal layer. The red arrowheads point to luminal cells, while the black arrowheads point to basal cells. Bars: 50µm. **b.** Immunohistochemistry analysis using a dual mouse and human anti-CDK12 antibody (see Methods) showing the endogenous CDK12 levels in the mammary gland of WT mice and the transgenic CDK12 expression in preneoplastic and overtly neoplastic lesions of the CDK12-KI mammary gland. A BT474 human cell line xenograft was used as a control for CDK12 overexpression in human breast cancers. Bars, 100 µm. **c.** Representative IHC images of KI67 expression in the mammary gland of CDK12-KI vs. WT female adult mice (age, 12-16 weeks). Bars, 50 µm. **d.** Left, Representative images of spheroids generated by CDK12-KI vs. WT MECs grown in suspension. Middle, Quantitative analysis of the spheroid-forming efficiency (SFE) displayed by CDK12-KI vs. WT MECs over serial passages in suspension culture conditions. The SFE was calculated relative to WT MECs at the first passage. Right, analysis of spheroid size. Data are means ± SD and statistical significance was evaluated via two sided unpaired *t* test (n=5) relative to WT in each condition. **, $P$=0.001; ***, $P$<0.001. Source data are provided as Source Data file.

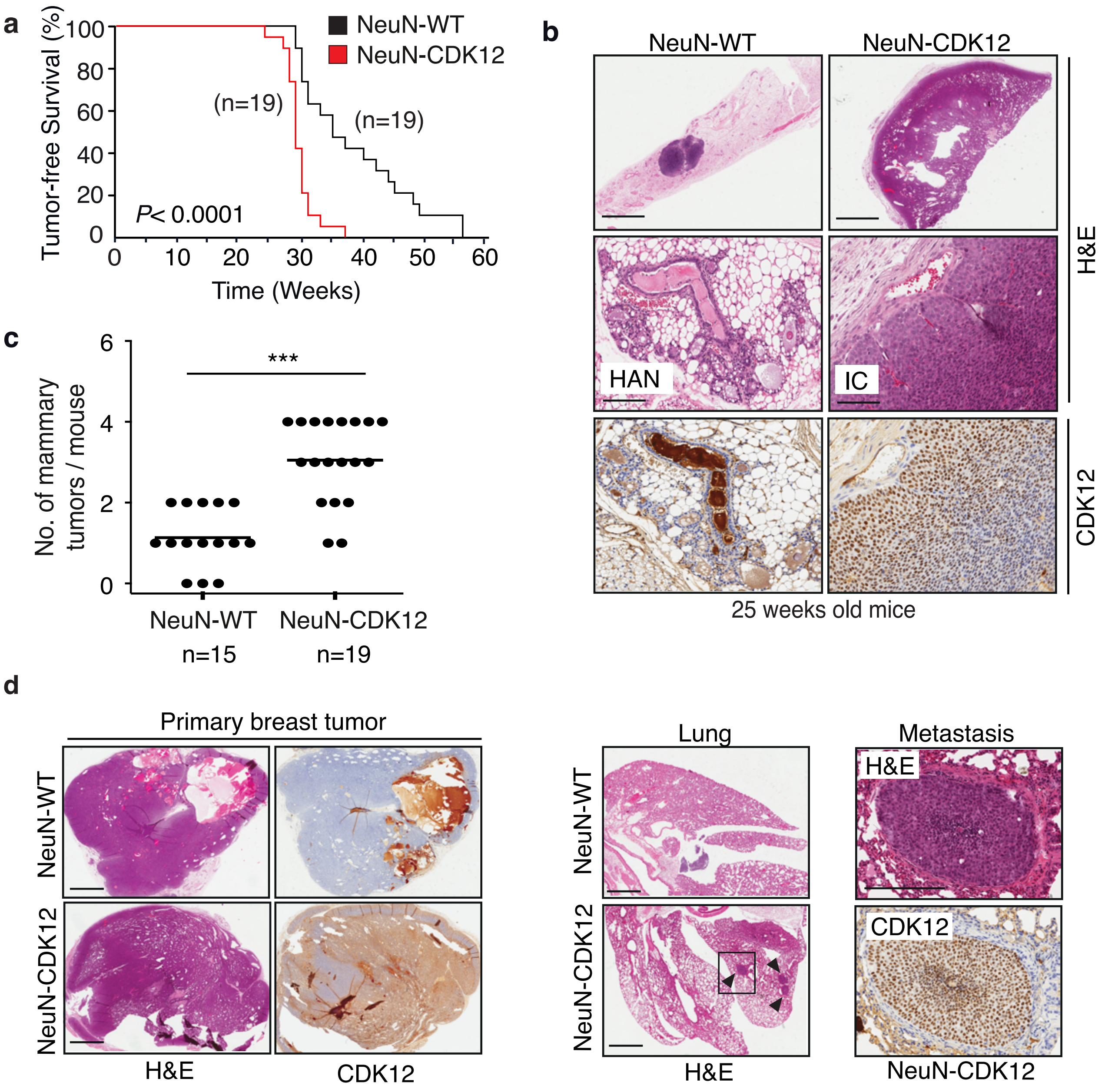


e

Incidence of pulmonary
Metastases at endpoint (%)

| Genotype | Incidence | *P* |
|---|---|---|
| NeuN-WT | 30.8% (12/39) | 0.0005 |
| NeuN-CDK12 | 85.7% (12/14) | |

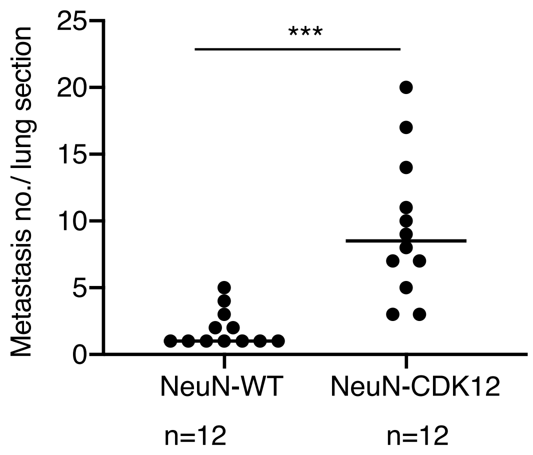


Supplementary Figure 2

**Supplementary Figure 2. CDK12 overexpression increases tumor onset and multiplicity and metastatic spreading in NeuN oncogene-driven mammary tumorigenesis.**

**a.** Kaplan-Meier analysis of tumor-free survival of adult female NeuN mice crossed with CDK12-KI (NeuN-CDK12) or WT (NeuN-WT) mice. Both groups are heterozygous for the Cre recombinase transgene. Mice were sacrificed when the maximum tolerable tumor burden was reached (~1.2 cm). Number (n) of mice, n=19 in each group. *P*<0.0001, long-rank test. **b.** Representative images from the histological analysis of the mammary glands of NeuN-CDK12 vs. NeuN-WT mice sacrificed at 25 weeks of age. H&E, hematoxylin and eosin staining; CDK12, anti-CDK12 IHC. HAN, hyperplastic alveolar nodule; IC, infiltrating carcinoma. Bars, 2 mm (top images); 100 µm (middle/bottom images). **c.** Number of mammary tumors/mouse detected in individual NeuN-CDK12 (n=19) vs. NeuN-WT (n=15) mice at the time of sacrifice. Statistical significance was evaluated via two sided unpaired *t* test. ***, *P*<0.001. **d.** Left, Representative images of primary mammary tumors in NeuN-CDK12 and NeuN-WT mice. Bars, 1.5 mm. Middle, Representative images of lung sections from NeuN-WT (top) and NeuN-CDK12 (bottom) mice. Arrowheads indicate representative multiple synchronous metastatic lesions in the lung of NeuN-CDK12 mice. Bars, 1.5 mm. The boxed area is magnified on the right. Bar, 250 µm. H&E, hematoxylin and eosin staining; CDK12, anti-CDK12 IHC. **e.** Left, Lung metastases incidence in NeuN-CDK12 vs. Neu-WT mice. Statistical significance, two tailed Fisher's test. *P*=0.0005*, P* value. Right, Quantification of metastases in lung sections from Neu-CDK12 vs. Neu-WT mice as in (e). Statistical significance, two sided unpaired *t* test. ***, *P*<0.001. Source data are provided as Source Data file.

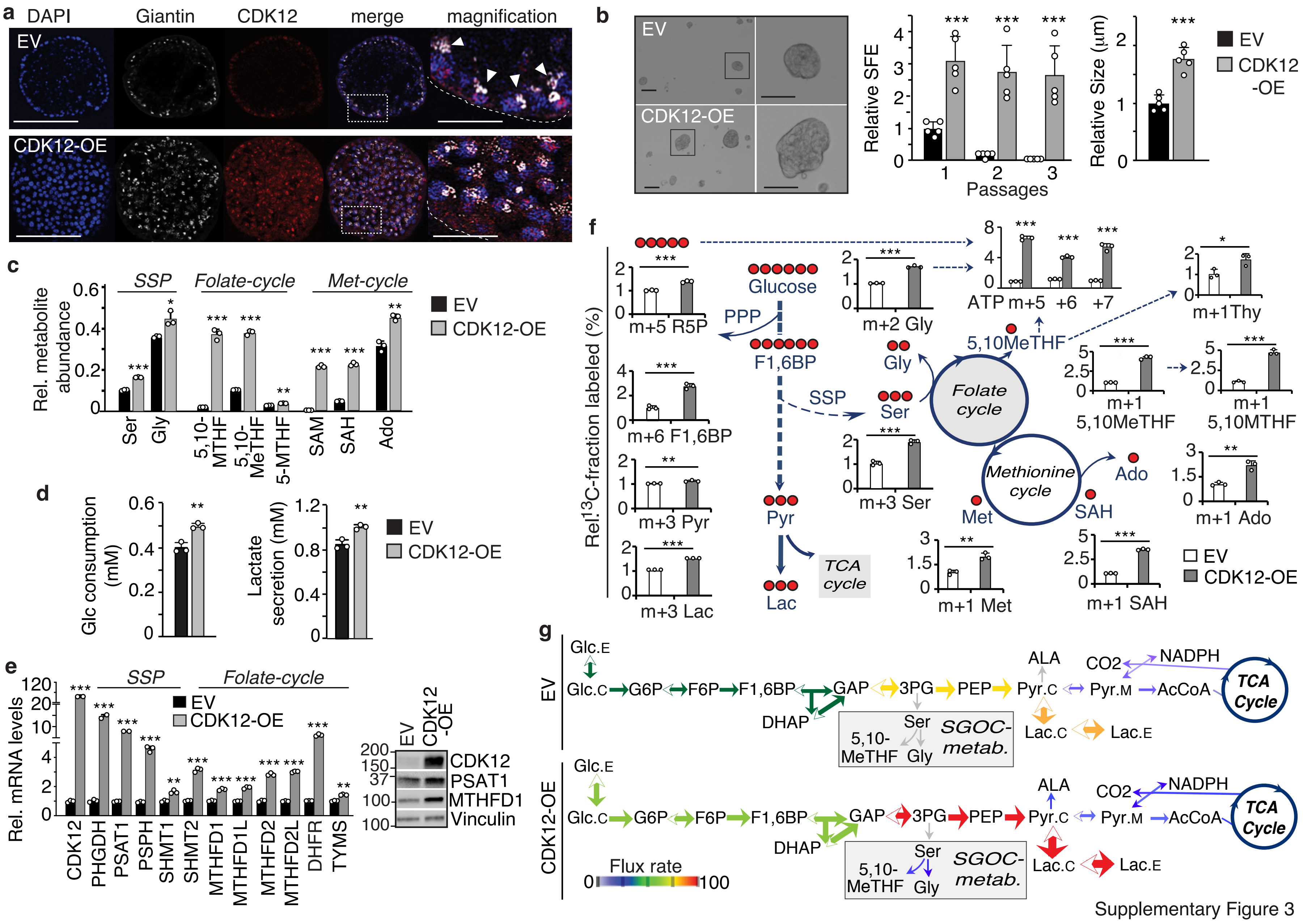


Supplementary Figure 3

**Supplementary Figure 3. CDK12 overexpression induces aberrant functional and metabolic phenotypes in normal mammary MCF10 cells.**

**a.** Confocal immunofluorescence characterization of the apical-basal polarity marker, Giantin, in CDK12-OE vs. EV MCF10A 3D-Matrigel acini. In these conditions, EV-MCF10A cells generated fully polarized acinar structures whereas CDK12-OE organoids were disorganized, filled and not polarized, as visualized by the aberrant Golgi position (Giantin). Dashed boxed areas in the merge fields are magnified on the right. Arrowheads in magnification show the normal apical-basal distribution of Giantin in control EV-MCF10A organoids. Bar, 100 µm; in magnified panels, 30 µm. DAPI (blue); Giantin (white); CDK12 (red). **b.** CDK12-OE and EV MCF10A cells were assayed for their ability to sustain multiple generations of spheres in suspension. Left, Representative images of spheroids generated by CDK12-OE vs. EV MCF10A cells. Dashed boxed areas are magnified on the right. Bars, 100 µm. Right, quantitation of the experiment showing the number of spheres generated in serial passages by CDK12-OE cells relative to control cells, and the average sphere size in the different conditions. SFE, sphere-forming efficiency. Data are means ± SD, and significance was evaluated via two sided unpaired *t* test (n=5 biological independent experiments). ****P*<0.001 vs. EV. **c.** Relative abundance of the indicated metabolites of SSP, folate and methionine cycle in CDK12-OE vs. EV MCF10A cells identified in LC-MS metabolomics (n=3 biological independent experiments, see abbreviations in Supplementary Table 1). Data are expressed relative to the internal standard (reserpine) and are means ± SD. Significance was evaluated via two sided unpaired *t* test. *, *P*=0.012; **, *P*=0.001; ***, *P*<0.001. **d.** Effect of CDK12 overexpression on extracellular lactate and glucose consumption levels**.** Lactate and glucose concentration in the extracellular medium of CDK12-OE vs. EV MCF10A cells at 48 hours after cell seeding, was measured by YSI biochemical analyzer (see Methods for details). Lactate and glucose levels are reported as mmol/L per $10^5$cells and are mean ± SD (n=3 biological independent experiments). ***P*= 0.003 (Glc consumption) and *P*= 0.004 (Lactate secretion) by two sided unpaired *t*-test. **e.** Left, RT-qPCR analysis for the indicated

enzymes of the serine synthesis pathway (SPP) and one-carbon pathway (Folate cycle) identified in the RNA-seq data (n=2) from CDK12-OE vs. EV MCF10A cells. Data are represented as relative change in mRNA levels in CDK12-OE vs. control cells. Bars represent the mean ± SEM from one representative experiment run in triplicate, out of three. **, $P<0.01$; ***, $P<0.001$ by two sided unpaired *t*-test. Results from two additional RT-qPCR experiments, each run in triplicate, are provided in Source Data file. Right, Immunoblot analysis of the expression of the indicated enzymes in CDK12-OE or EV MCF10A cells. Vinculin, loading control. Uncropped blots in Source Data file. **f.** Schematic of derivation and contribution of carbon atoms from the glucose backbone (red, carbon atoms resourced from the $^{13}$C-glucose) into the pentose phosphate pathway (PPP), serine synthesis pathway (SSP), folate and methionine cycles by LC-MS analysis of CDK12-OE vs. EV MCF10A. Bar graphs show mass isotopologue distribution of the indicated metabolites measured in CDK12-OE *vs.* EV MCF10A grown in medium containing $^{13}$C-glucose for 48hr. Data are mean ± SD, and expressed relative to EV. Significance was evaluated via two sided unpaired *t*-test (n=3). *, $P=0.026$; **, $P<0.01$;***, $P<0.001$ vs. EV. **g.** Quantitative metabolic flux analysis**.** Schematic of metabolic flux distribution in the central metabolic pathways of glycolysis, SGOC metabolism (SPP and one-carbon) and TCA cycle in CDK12-OE vs. EV MCF10A grown in $^{13}$C-glucose. Arrow thickness and color codes reflect the log2 fold change of the reaction rates in the CDK12-OE vs. EV MCF10A and are proportional to the net flux rate. Reactions that are reversible are shown with two-headed arrows (see abbreviations in Supplementary Table 1). E, extracellular; C, cytosolic; M, mitochondrial. Statistics on the estimated reaction rates are presented in Supplementary Data 3. Source data are provided as Source Data file.

**a**

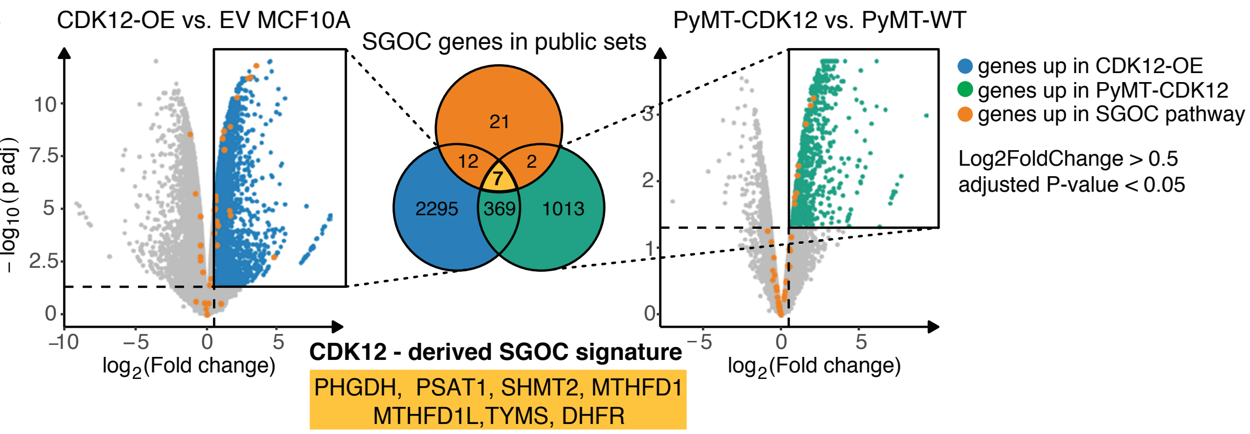


**b** Integrative IHC vs. RT-qPCR analysis

| CDK12 protein | mRNA low | mRNA high | Total |
|---|---|---|---|
| not-overexpr. | 418 (31.5%) | 907 (68.5%) | 1325 (77.4%) |
| overexpressed | 43 (11.1%) | 345 (88.9%) | 388 (22.6%) |
| Total | 461(26.9%) | 1252(73.1%) | 1713 |

| CDK12 status | Patient No. | % |
|---|---|---|
| HIGH | 388 | 22.6 |
| LOW | 907 | 53 |
| NULL | 418 | 24.4 |
| Total | 1713 | |

**c**

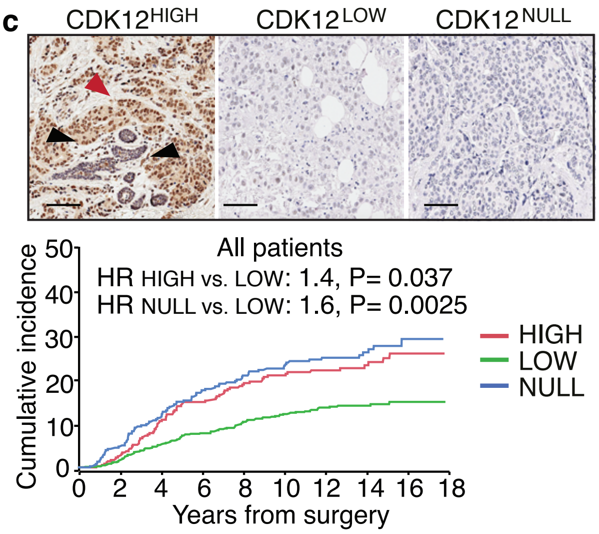


**d** Integrative IHC vs. RT-qPCR analysis

HER2-neg. patients

| CDK12 protein | mRNA low | mRNA high | Total |
|---|---|---|---|
| not-overexpr. | 384 (30.9%) | 860 (69.1%) | 1244 |
| overexpressed | 33 (15.1%) | 185 (84.9%) | 218 |
| Total | 417 | 1045 | 1462 |

| CDK12 status | Patient No. | % |
|---|---|---|
| HIGH | 218 | 15 |
| LOW | 860 | 58.8 |
| NULL | 384 | 26.2 |
| Total | 1462 | |

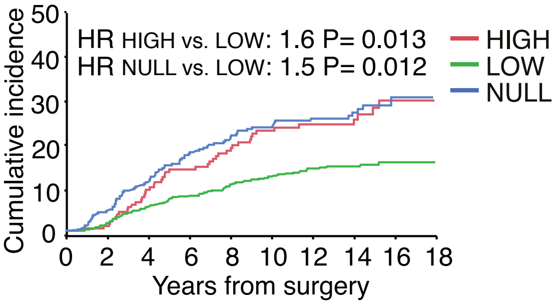


**e** Integrative IHC vs. RT-qPCR analysis

HER2-pos. patients

| CDK12 protein | mRNA low | mRNA high | Total |
|---|---|---|---|
| not-overexpr. | 25 (47.2%) | 28 (52.8%) | 53 |
| overexpressed | 8 (5.1%) | 148 (94.9%) | 156 |
| Total | 33 | 176 | 209 |

| CDK12 status | Patient No. | % |
|---|---|---|
| HIGH | 156 | 74.6 |
| LOW | 28 | 13.4 |
| NULL | 25 | 12 |
| Total | 209 | |

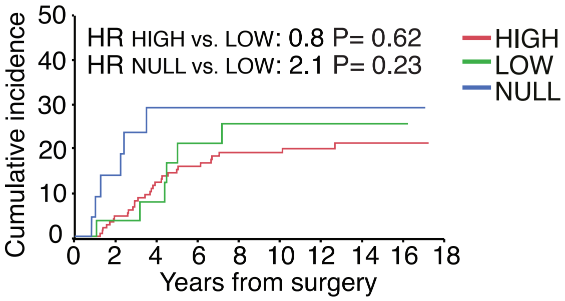


Supplementary Figure 4

**Supplementary Figure 4. Clinical relevance of CDK12 overexpression to real life human breast cancer patients**.

**a.** Schematic representation of the derivation of the $SGOC_{CDK12}$ signature. Two independent lists of differentially regulated genes (DEGs) (log2 fold change ≥0.5, adjusted *P*-value < 0.05) were obtained from the comparative transcriptomic analysis of human CDK12-OE vs. EV MCF10A cells and mouse PyMT/CDK12 vs. PyMT/WT cells (see also Supplementary Data 1). Upregulated genes are reported as blue and green dots in the Volcano plots relative to the comparison of CDK12-OE vs. EV MCF10A cells (left) and PyMT-CDK12 vs. PyMT-WT cells (right), respectively, with not upregulated represented as gray dots in either comparison. Shown in the Venn diagram in the middle is the intersection of these two lists of CDK12-related upregulated genes with an integrated list of 42 genes annotated in SGOC pathways in the following public pathway databases: KEGG-One Carbon Pool By Folate, KEGG-Folate Biosynthesis, REACTOME-Metabolism Of Folate And Pterines, REACTOME-Serine Biosynthesis (indicated above the Venn diagram). The genes of this 42 gene list and their distribution across DEGs and not DEGs in the comparison of CDK12-OE vs. EV MCF10A cells and PyMT-CDK12 vs. PyMT-WT cells are represented as orange dots in the respective Volcano plots. By this integrative approach, a signature of 7 CDK12-upregulated key SGOC genes ($SGOC_{CDK12}$ signature) was identified: DHFR, TYMS, MTHFD1L, MTHFD1, PHGDH, SHMT2 and PSAT1. The numbers in overlapping arcs indicate the number of genes shared between and among the three lists. **b.** Top, Distribution of patients with overexpressed or not-overexpressed (not-overexpr.) CDK12 protein (by IHC) and mRNA (by RT-qPCR) levels in a consecutive cohort of 1713 breast cancer patients (see Methods for details). Bottom, Integration of IHC and RT-qPCR data allowed the identification of 3 classes of patients with HIGH, LOW or NULL CDK12 status. The number (Patient No.) and percentage (%) of patients in each class is shown. **c.** Top, Representative IHC images of $CDK12^{HIGH}$, $CDK12^{LOW}$ and $CDK12^{NULL}$ breast tumors. The red arrowhead indicates a tumor area with CDK12 overexpression. Note that CDK12 IHC staining in the residual areas of

normal mammary gland (indicated by black arrowheads) is indistinguishable from that of CDK12$^{LOW}$ (middle panel) and CDK12$^{NULL}$ (right panel) tumors. Bars, 200 µm. Bottom, Cumulative incidence of distant metastasis in the entire cohort of breast cancer patients stratified by CDK12 status as in (b). HR, multivariable hazard ratio adjusted for ER/PgR, HER2, Grade, pT, pN, KI67, age. *P*, *P*-value, by Wald-test. **d.** Top, Distribution of patients with overexpressed or not-overexpressed (not-overexpr.) CDK12 protein (by IHC) and mRNA (by RT-qPCR) levels in the subgroups of HER2-negative (HER2-neg.) breast cancer patients of the cohort described in (b). Middle, Table showing the number (Patient No.) and percentage (%) of patients included in each CDK12 class (HIGH, LOW or NULL) based on integration of IHC and RT-qPCR data. Bottom, Cumulative incidence of distant metastasis in HER2-neg. breast cancer patients stratified by CDK12 status. HR, multivariable hazard ratio. *P*, *P*-value, Wald-test. **e.** Top, Distribution of patients with overexpressed or not-overexpressed (not-overexpr.) CDK12 protein (by IHC) and mRNA (by RT-qPCR) levels in the subgroups of HER2-positive (HER2-pos.) breast cancer patients of the cohort described in (b). Middle, Table showing the number (Patient No.) and percentage (%) of patients included in each CDK12 class (HIGH, LOW or NULL) based on integration of IHC and RT-qPCR data. Bottom, Cumulative incidence of distant metastasis in HER2-pos. breast cancer patients stratified by CDK12 status. HR, multivariable hazard ratio. *P*, *P*-value, Wald-test. Source data are provided as Source Data file.

**a** HER2-negative patients

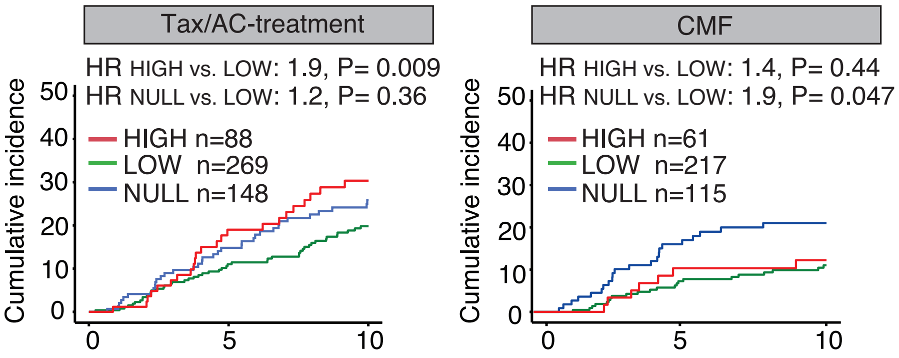


**b** All patients: Adjuvant CT (trial IBCSG 22-00)

| CDK12 (IHC) | Treatment groups | No. at risk (No. of events) | | | | Total events |
|---|---|---|---|---|---|---|
| LOW | CTR | 73(0) | 5(13) | 35(13) | 7(13) | 13 |
| | CM | 74(0) | 56(13) | 44(14) | 15(14) | 14 |
| HIGH | CTR | 31(0) | 22(7) | 15(8) | 1(8) | |
| | CM | 35(0) | 27(0) | 19(1) | 3(1) | |
| Years from random assignment | | 0 | 5 | 10 | 15 | |

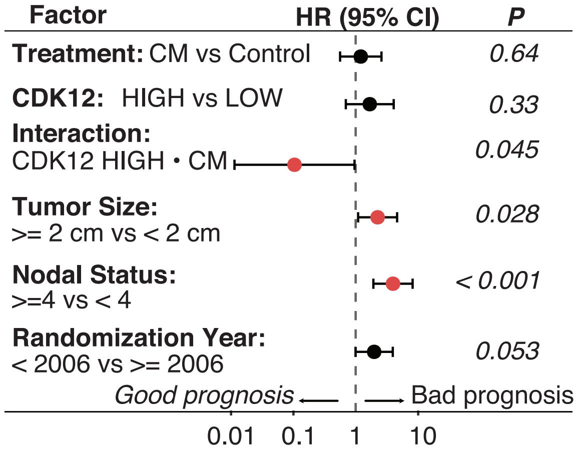


**c** Neoadjuvant treated patients

| | PCR | CDK12 protein (IHC) low | high | Total | OR PCR | 95% CI | *P* |
|---|---|---|---|---|---|---|---|
| Luminal | no PCR | 37 (37.4%) | 62 (62.6%) | 99 | 5.4 | 1.8-15.8 | 0.001 |
| | PCR | 16 (76.2%) | 5 (23.8%) | 21 | | | |
| | Total | 53 (44.2%) | 67 (55.8%) | 120 | | | |
| TNBC | no PCR | 19 (39.6%) | 29 (60.4%) | 48 | 2.8 | 1.2-6.3 | 0.012 |
| | PCR | 33 (64.7%) | 18 (35.3%) | 51 | | | |
| | Total | 52 (52.5%) | 47 (47.5%) | 99 | | | |
| HER2+ | no PCR | 7 (17.1%) | 34 (82.9%) | 41 | 4.2 | 1.1-16.2 | 0.040 |
| | PCR | 6 (46.1%) | 7 (53.9%) | 13 | | | |
| | Total | 13 (24.1%) | 41 (75.9%) | 54 | | | |

**d** Tax/AC-resistant TNBC patients treated with 2nd line CMF

| TNBC | CDK12 protein (IHC) low | high | Total | *P* |
|---|---|---|---|---|
| Death | 27 (62.8%) | 27 (32.9%) | 54 (43.2%) | 0.002 |
| No Death | 16 (37.2%) | 55 (67.1%) | 71 (56.8%) | |
| Total | 43 (34.4%) | 82 (65.6%) | 125 | |

**e** Distant metastasis prognostication and chemotherapy prediction in patients stratified by CDK12 status

| CDK12 molecular classes | Prognosis in unstratified patients | Prediction of clinical outcome by type of CT treatment: Tax/AC | CMF |
|---|---|---|---|
| $CDK12^{HIGH}$ | Unfavorable | Unfavorable | Favorable |
| $CDK12^{LOW}$ | Favorable | Favorable | Favorable |
| $CDK12^{NULL}$ | Unfavorable | Unfavorable | Unfavorable |

Supplementary Figure 5

**Supplementary Figure 5. CDK12 overexpression is a therapeutic biomarker in breast cancer patients.**

**a.** Kaplan-Meier analysis of the 10-year cumulative incidence of distant metastasis in HER2-neg. patients stratified by CDK12 status (as described in Supplementary Fig. 4b, c) in response to different types of adjuvant chemotherapy (CT): methotrexate-based (CMF, cyclophosphamide, methotrexate, fluorouracil) CT (top) or taxane and anthracycline (Tax/AC)-based CT (bottom). The patient number in each class (n), the HR, multivariable hazard ratio (any time) and the *P* value (Wald-test) are indicated. **b.** Top, Description of the cohort of 213 adjuvant patients from the randomized phase III IBCSG 22-00 trial, stratified by CDK12 IHC status (LOW vs. HIGH) and analyzed for their response to metronomic maintenance chemotherapy (CT) with cyclophosphamide+methotrexate (CM) or no treatments (CTR). Endpoint: Cumulative Incidence of distant metastasis. Number at risk and number of events according to the years from random group assignment are indicated. Bottom, Forest plot showing multivariable hazard ratios (HR), 95% confidence intervals (CI) and *P* values (Wald-test) for the association between the indicated factors and good (HR<1) or bad (HR>1) prognosis in patients stratified by their CDK12 (HIGH vs. LOW) status, by Cox proportional hazards model. This analysis shows that the association between high CDK12 status, CM therapy and more favorable prognosis is independent of the number of positive lymph nodes and size of the tumor, which are the major prognostic factors. **c.** Description of the luminal, triple-negative (TNBC) and HER2-positive (HER2+) patient cohorts stratified by CDK12 IHC status (LOW vs. HIGH) and analyzed for their response to neoadjuvant treatments. Endpoint: pathological complete response (PCR, i.e., complete absence of residual infiltrating cancer cells) vs. no-PCR (including stable disease, disease progression and partial objective response). OR, odds ratio. Confidence intervals (CI) and *P* values (Likelihood ratio test) are indicated. **d.** Description of the cohort of primary taxane/anthracycline (Tax/AC) chemotherapy-resistant TNBC patients treated with salvage MTX-based chemotherapy. Endpoint: Cumulative Incidence of Death. *P, P* value (two tailed Fisher's test). Source data are provided as

Source Data file. **e.** Schematic overview of the clinical behavior of CDK12 as a prognostic and therapy predictive biomarker, summarizing the results of the clinical analyses in breast cancer patients stratified according to their CDK12 status ($CDK12^{HIGH}$, $CDK12^{LOW}$ ,$CDK12^{NULL}$) described in Fig. 8. The table shows that patients with a $CDK12^{HIGH}$ status, who typically display a poor prognosis, maintain a very aggressive disease course in response to treatment with cytotoxic Tax/AC chemotherapy, indicative of a substantial refractoriness of these patients to this treatment, but revert to a favorable prognosis in response to methotrexate-based treatments, indicative of their selective vulnerability to targeted antimetabolite therapy interfering with the pathological SGOC metabolism. In contrast, no substantial differences are observed in $CDK12^{LOW}$ and $CDK12^{NULL}$ patients, who maintain a favorable or unfavorable prognosis, respectively, independently of the type of chemotherapy with Tax/AC or MTX-based regimens. The reversion of the poor prognostic outcome of $CDK12^{HIGH}$ patients to a favorable disease course in response to MTX-based regimens vis a vis the absence of any effect of this type of antimetabolite therapy in $CDK12^{LOW}$ and $CDK12^{NULL}$ patients reveals a typical oncogene addiction-like therapeutic vulnerability in CDK12-overexpressing breast cancers.

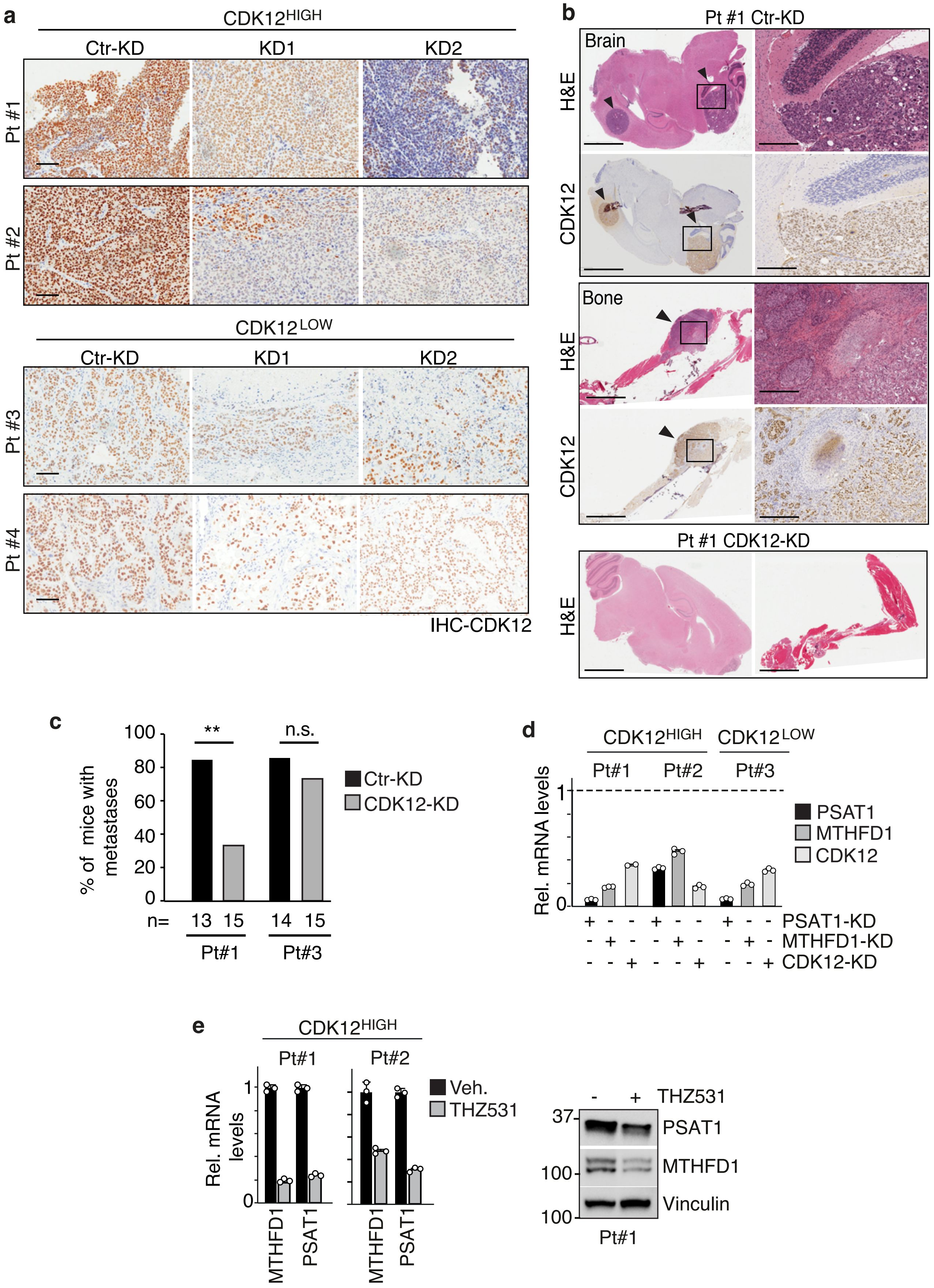


Supplementary Figure 6

**Supplementary Figure 6. Targeting CDK12-induced alterations of the SGOC network impairs the tumorigenic and metastatic potential of CDK12-overexpressing human breast cancers.**

**a.** Representative IHC images of CDK12 expression in PDXs obtained from CDK12$^{HIGH}$ (Pt#1 and #2) and CDK12$^{LOW}$ (Pt#3 and #4) breast cancer patients, silenced or not for CDK12 expression (CDK12-KD and Ctr-KD, respectively) as in Figure 5a. Bars, 200 µm. **b.** Representative images of brain and bone metastases in mice intracardiacally injected with control-silenced (Ctr-KD) vs. CDK12-silenced (CDK12-KD) cells from a CDK12$^{HIGH}$ patient (Pt #1). Arrowheads indicate the presence of brain (top panels) or bone (middle panels) metastases in mice injected with Pt#1 Ctr-KD cells. Boxed areas are magnified on the right. Bottom panels show absence of metastases in brain (left) and bone (right) tissues from mice injected with Pt#1 CDK12-KD cells. H&E, hematoxylin and eosin; CDK12, CDK12 IHC staining. Bars, 3 mm (left panels); 100 µm (in magnifications). **c.** Shown are the percentages of mice displaying distant organ metastases (brain+bone) after intracardiac injection of Ctr-KD vs. CDK12-KD cells from a CDK12$^{HIGH}$ (Pt#1) and a CDK12$^{LOW}$ (Pt#3) PDX at a fixed time-point (6 weeks post-injection). The number of mice in each group is indicated. **, *P*=0.009, two tailed Fisher's test; n.s., not significant. **d.** RT-qPCR showing efficacy of silencing of PSAT1, MTHFD1 and CDK12 in CDK12$^{HIGH}$ (Pt#1 and #2) or CDK12$^{LOW}$ (Pt#3) PDX cells. Data are expressed as the mean ± SEM, relative to control-silenced cells (Ctr-KD=1, indicated by horizontal dashed line) for each condition. **e.** Left, RT-qPCR analysis of the indicated SGOC network genes in CDK12$^{HIGH}$ PDX cells (Pt#1 and #2) treated with THZ531 (100 nM) or control-treated (Veh.) for 48 h. Data are reported as relative change in mRNA levels in THZ531- vs. Veh.-treated cells. Bars represent the mean ± SEM. Data are from one representative experiment, out of two. Right, Immunoblot analysis of the indicated SGOC enzyme levels in CDK12$^{HIGH}$ PDX cells (Pt#1), treated or not with 100 nM THZ531 for 48 h. Vinculin, loading control. Uncropped blot in Source Data. Source data are provided as Source Data file.

**a**

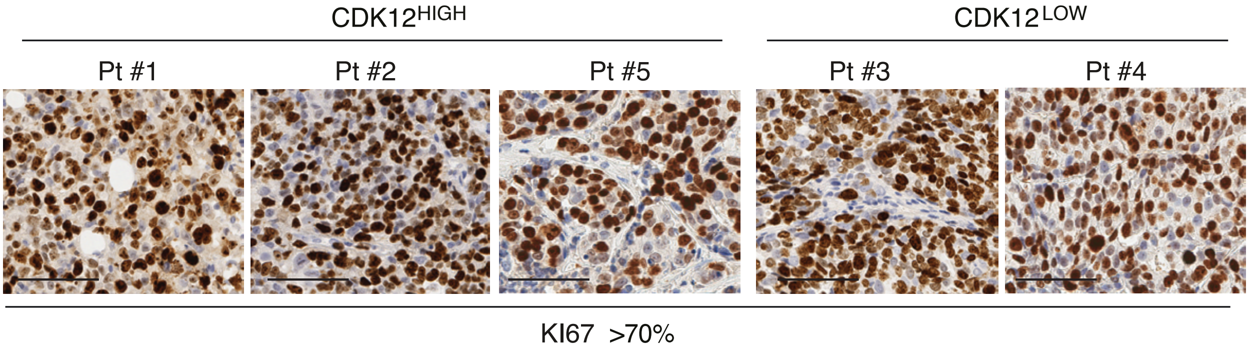


**b**

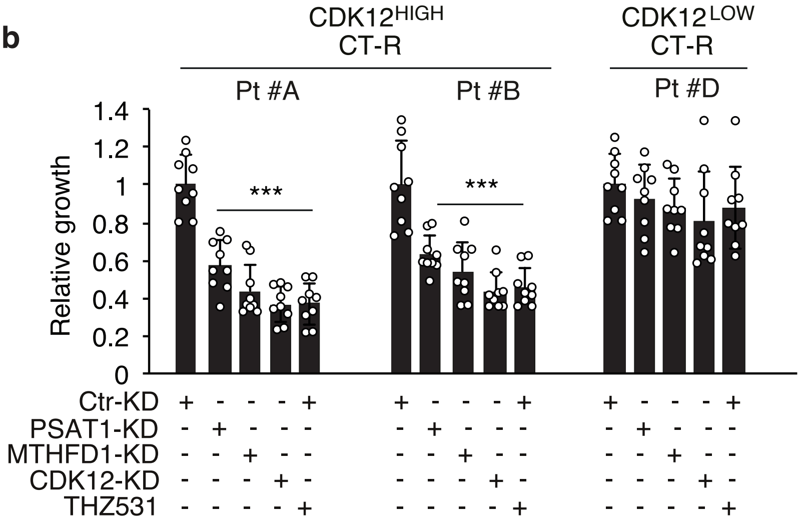


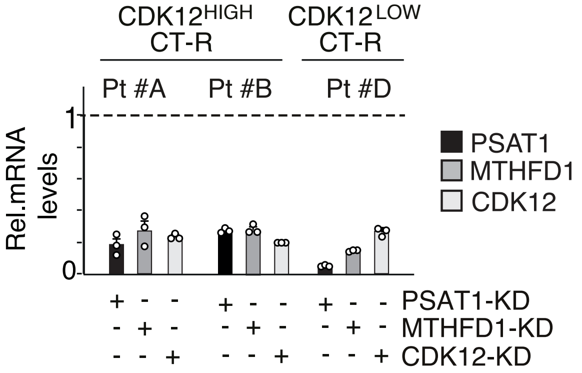


Supplementary Figure 7

**Supplementary Figure 7. CDK12-induced alterations of the SGOC network represent an actionable therapeutic target in otherwise chemoresistant CDK12-overexpressing human breast cancers.**

**a.** Representative IHC images for the proliferation marker, KI67, in CDK12$^{HIGH}$ (Patient #1, #2, #5) vs. CDK12$^{LOW}$ (Patient #3, #4) PDXs. The percentage of KI67 positive cells is indicated. Bars, 100μm. **b.** Top, Three-day proliferation of CT-R cells from CDK12$^{HIGH}$ (Patients #A and #B) vs. CDK12$^{LOW}$ (Patient #D) PDXs control-silenced or silenced for PSAT1 (PSAT1-KD), MTHFD1 (MTHFD1-KD) and CDK12 (CDK12-KD), or treated with 100 nM THZ531. Data are from three independent experiments and expressed as the mean ± SD relative to matching Ctr-KD. ***, $P<0.001$ by two sided unpaired *t*-test. Bottom, RT-qPCR analysis showing silencing efficacy for the indicated genes. Data are expressed as as the mean ± SEM relative to control-silenced cells (Ctr-KD=1, indicated by horizontal dashed line) for each condition. Source data are provided as Source Data file.

**SUPPLEMENTARY TABLE 1**

**List of abbreviations relative to Figure 4b**

| **Abbreviated Hits** | **Description** |
|---|---|
| SAH | S-Adenosylhomocysteine |
| G6P | Glucose 6 phosphate |
| F6P | Fructose 6 phosphate |
| 5,10 MTHF | 5,10-Methylenetetrahydrofolic acid |
| 5,10 MeTHF | 5,10-Methenyltetrahydrofolic acid |
| 5-MTA | 5-Methylthioadenosine |
| SAM | S-Adenosylmethionine |
| Glu-5-P | Glutamic acid 5-phosphate |
| F 1,6 BP | Fructose 1,6-bisphosphate |
| GluSA | Glutamic gamma-semialdehyde |
| GDP | Guanosine diphosphate |
| ADP | Adenosine diphosphate |
| AMP | Adenosine monophosphate |
| Succ SA | Succinic acid semialdehyde |
| 2-HG | 2-Hydroxyglutaric acid |
| GMP | Guanosine monophosphate |

**List of abbreviations relative to Supplementary Figure 3c**

| Metabolite | Description |
|---|---|
| Ser | Serine |
| Gly | Glycine |
| 5,10-MTHF | 5,10-Methylenetetrahydrofolic acid |
| 5,10-MeTHF | 5,10-Methenyltetrahydrofolic acid |
| 5-MTHF | 5-Methyltetrahydrofolic acid |
| SAM | S-Adenosylmethionine |
| SAH | S-Adenosylhomocysteine |
| S-Mado | S-methyladenosine |
| Ado | Adenosine |

**List of abbreviations relative to Supplementary Figure 3g**

| | |
|---|---|
| GLC.E | Glucose (Extracellular) |
| GLC.C | Glucose (Cytosolic) |
| G6P | Glucose 6 phosphate |
| F6P | Fructose 6 phosphate |
| F1,6BP | Fructose 1,6-bisphosphate |
| DHAP | Dihydroxyacetone phosphate |
| GAP | Glyceraldehyde 3-phosphate |
| 3PG | 3-phosphoglycerate |
| PEP | Phosphoenolpyruvate |
| PYR.C | Pyruvic acid (Cytosolic) |

| | |
|---|---|
| ALA | Alanine |
| LAC.C | Lactic Acid (Cytosolic) |
| LAC.E | Lactic Acid (Extracellular) |
| PYR.M | Pyruvic acid (Mitochondrial) |
| AcCoA | Acetyl-CoA |
| CO2 | Carbon Dioxide |
| NADPH | Nicotinamide Adenine Dinucleotide Phosphate Hydrogen |
