# Supplementary material for: CDK12 promotes tumorigenesis but induces vulnerability to therapies inhibiting folate one-carbon metabolism in breast cancer
Source: Nat Commun. 2022 May 12;13:2642. doi: 10.1038/s41467-022-30375-8 (PMC9098894; doi:10.1038/s41467-022-30375-8)
Supplement: Supplementary file 3 — Description of Additional Supplementary Files [file 41467_2022_30375_MOESM3_ESM.pdf]

DESCRIPTION OF ADDITIONAL SUPPLEMENTARY FILES

Supplementary Data 1.

This table contains the list of genes differentially regulated in the global transcriptomic profile of CDK12-OE vs. control EV MCF10A cells (MCF10A_CDK12-OE_vs_EV sheet) and CDK12-KI/PyMT vs. WT/PyMT tumor cells (PyMT_CDK12-KI_*vs*_PyMT_WT sheet). For each entry, the following information is reported (from left to right):

- Gene symbol (gene symbol corresponding to HUGO nomenclature).

- log2(fold change) of gene transcriptional levels in CDK12 cells compared to control samples;

- logCPM (average logarithm of counts per million reads);

- *P* value for differential gene expression resulting from quasi-likelihood F-tests;

- FDR adjusted *P* value with Benjamini Hochberg method;

- gene type (i.e. coding, non-coding);

- descriptive gene name;

- Ensembl gene accession number;

- Entrez gene accession number.

Statistical analyses were performed using the edgeR package of R software.

Supplementary Data 2.

This table contains the list of metabolites differentially regulated in the untargeted metabolomics analysis of CDK12-OE vs. control EV MCF10A cells by LC-MS. For each entry, the following information is reported (from left to right):

- Hit (metabolite name corresponding to KEGG nomenclature);

- T.stat (T-Statistics, cutoff>1.5 for significantly higher metabolite abundance in CDK12-OE vs. EV MCF10A cell comparison, across all samples;

- *P* value and -log10(P) (Negative of the base 10 logarithm of *P* values); FDR, False Discovery Rate (FDR-adjusted *P* value threshold to 0.25); Statistical analyses were performed using the MetaboAnalyst 3.0 tool.

Supplementary Data **3.** MFA flux analysis**.**
