# Supplementary material for: CDK12 promotes tumorigenesis but induces vulnerability to therapies inhibiting folate one-carbon metabolism in breast cancer
Source: Nat Commun. 2022 May 12;13:2642. doi: 10.1038/s41467-022-30375-8 (PMC9098894; doi:10.1038/s41467-022-30375-8)
Supplement: Supplementary file 7 — Reporting Summary [file 41467_2022_30375_MOESM7_ESM.pdf]

nature portfolio

Corresponding author(s): Salvatore Pece and Daniela Tosoni

Last updated by author(s): Feb 25, 2022

Reporting Summary

Nature Portfolio wishes to improve the reproducibility of the work that we publish. This form provides structure for consistency and transparency in reporting. For further information on Nature Portfolio policies, see our Editorial Policies and the Editorial Policy Checklist.

Statistics

For all statistical analyses, confirm that the following items are present in the figure legend, table legend, main text, or Methods section.

n/a	Confirmed	
☐	☒	The exact sample size (n) for each experimental group/condition, given as a discrete number and unit of measurement
☐	☒	A statement on whether measurements were taken from distinct samples or whether the same sample was measured repeatedly
☐	☒	The statistical test(s) used AND whether they are one- or two-sided<br>*Only common tests should be described solely by name; describe more complex techniques in the Methods section.*
☐	☒	A description of all covariates tested
☐	☒	A description of any assumptions or corrections, such as tests of normality and adjustment for multiple comparisons
☐	☒	A full description of the statistical parameters including central tendency (e.g. means) or other basic estimates (e.g. regression coefficient) AND variation (e.g. standard deviation) or associated estimates of uncertainty (e.g. confidence intervals)
☐	☒	For null hypothesis testing, the test statistic (e.g. F, t, r) with confidence intervals, effect sizes, degrees of freedom and P value noted<br>*Give P values as exact values whenever suitable.*
☒	☐	For Bayesian analysis, information on the choice of priors and Markov chain Monte Carlo settings
☒	☐	For hierarchical and complex designs, identification of the appropriate level for tests and full reporting of outcomes
☐	☒	Estimates of effect sizes (e.g. Cohen's d, Pearson's r), indicating how they were calculated

Our web collection on statistics for biologists contains articles on many of the points above.

Software and code

Policy information about availability of computer code

Data collection

- IHC images were collected with Aperio ScanScope (v 12.2.2 Leica Biosystem).
- Immuno Blotting acquisition were performed by films and/or with Image Lab software (v 5.2.1 Bio-Rad Laboratories).
- Immunofluorescence data was collected with Las X (v 3.5 Leica).
- RT-qPCR data were collected with 7500 Software version 2.3 (Applied Biosystems) or with LightCycler480 (Roche).
- Metabolomic experiments were collected with MassHunter ProFinder and MassHunter VistaFlux software (Agilent) or YSI2950 bioanalyzer (YSI Incorporated, Yellow Springs, OH, USA).
- NSG data were sequenced by Illumina NovaSeq 6000.

Data analysis	- IHC images were analyzed with Aperio ScanScope (v 12.2.2. Leica) and/or ImageJ (v1.52, NIH).<br>- 2D- and 3D- growth assays where analyzed with Excel (v17.0, Microsoft), JMP software (v14, SAS Institute), Prism (v8, Graph Pad Software) or https://www.graphpad.com/quickcalcs/contingency1.cfm.<br>- Analysis of in vivo experiments was performed with Excel (v17.0, Microsoft), JMP software (v14, SAS Institute), Prism (v8, Graph Pad Software)<br>- Metabolomic experiments were analyzed with MetaboAnalyst 3.0 tool or Excel (v17.0, Microsoft)<br>- NGS data were processed with NF-CORE RNASeq pipeline (https://github.com/nf-core/rnaseq, version 3.1) using the Salmon pseudo-aligner to quantify transcript abundances.<br>- Differential expression analysis of RNA sequencing experiments was performed using the edgeR package (version 3.34) in R (http://www.r-project.org, version 4.1).<br>- Survival analysis were performed with the R (http://www.r-project.org, version 4.0.2) and JMP (SAS institute, version 14.0) software.<br>- Statistical analysis of the METABRIC dataset were performed in R (http://www.r-project.org, version 4.1)<br>See Materials and Methods for details.

For manuscripts utilizing custom algorithms or software that are central to the research but not yet described in published literature, software must be made available to editors and reviewers. We strongly encourage code deposition in a community repository (e.g. GitHub). See the Nature Portfolio guidelines for submitting code & software for further information.

Data

Policy information about availability of data

All manuscripts must include a data availability statement. This statement should provide the following information, where applicable:

- Accession codes, unique identifiers, or web links for publicly available datasets
- A description of any restrictions on data availability
- For clinical datasets or third party data, please ensure that the statement adheres to our policy

The raw and processed RNA sequencing data generated in this study have been deposited in the GEO database under accession code GSE189274 [https://www.ncbi.nlm.nih.gov/geo/query/acc.cgi?acc=GSE189274].
The RNA expression and associated clinicopathological data from the METABRIC cohort used in this study are available in cBioPortal under accession id brca_metabric [https://www.cbioportal.org/study/summary?id=brca_metabric].
Human and mouse gene set annotations for pathway analysis and CDK12-SGOC signature definition were retrieved from MSigDB [https://www.gsea-msigdb.org/gsea/msigdb/] with the msigdbr package (version 7.4.1) in R.
Human GRCh38 and mouse GRCm39 reference genomes (primary genome assembly) used for the alignment of RNA sequencing data are available on GENCODE [https://www.gencodegenes.org/].
Publicly available RNA expression and associated clinicopathological data of 1904 patients from the METABRIC (Molecular Taxonomy of Breast Cancer Consortium) dataset 3 were retrieved from cBioPortal (http://www.cbioportal.org/).
All data are available in the manuscript, in the Supplementary Information or in the Source Data files.

Field-specific reporting

Please select the one below that is the best fit for your research. If you are not sure, read the appropriate sections before making your selection.

☒ Life sciences ☐ Behavioural & social sciences ☐ Ecological, evolutionary & environmental sciences

For a reference copy of the document with all sections, see nature.com/documents/nr-reporting-summary-flat.pdf

Life sciences study design

All studies must disclose on these points even when the disclosure is negative.

Sample size	No sample size calculations were performed; sample sizes were determined to be adequate based on the magnitude and consistency of measurable differences between groups. The number of experiments (n) or samples/tumors analyzed (N) is indicated in each figure legend.
Data exclusions	From a retrospective longitudinal cohort of 2,453 BC patients (IEO BC 97-00 cohort, previously described in Pece S. et al. EBioMedicine 2019; Schiano Lomoriello I. et al. Nat Commun. 2020), 2,275 patient samples were appropriate for the construction of ad hoc tissue microarray (TMAs): samples with massive inflammatory infiltration, massive necrosis, minimal areas of infiltrating carcinoma were discarded. Concomitant IHC and RT-qPCR data were available for a total of 1,713 patients, either because of loss of tissue cores during the IHC staining/lack of neoplastic tissue in the tissue core or because of spurious results in the RT-qPCR analysis (due to poor quality mRNA).<br>No exclusion criteria were applied for the cohort of patients enrolled at IEO from years 2000 to 2012 and randomized to metronomic CM vs. no therapy in the context of the international registered clinical trial, IBCGS22-00 (described in Supplementary Fig. 5b).
Replication	The number of experiments replicated (n) is indicated in each figure legend.
Randomization	Samples were randomly assigned.
Blinding	Investigators were blinded to group allocation during IHC analysis of specimens.<br>For the other experiments, the investigators were not blinded to experimental groups during data collection and analysis. Data reported in this latter case is not subjective.

Reporting for specific materials, systems and methods

We require information from authors about some types of materials, experimental systems and methods used in many studies. Here, indicate whether each material, system or method listed is relevant to your study. If you are not sure if a list item applies to your research, read the appropriate section before selecting a response.

Materials & experimental systems

n/a	Involved in the study
	☒ Antibodies
	☒ Eukaryotic cell lines
☒	☐ Palaeontology and archaeology
	☒ Animals and other organisms
☒	☐ Human research participants
	☒ Clinical data
☒	☐ Dual use research of concern

Methods

n/a	Involved in the study
☒	☐ ChIP-seq
☒	☐ Flow cytometry
☒	☐ MRI-based neuroimaging

Antibodies

Antibodies used

Primary Abs:
- Homemade mouse monoclonal antibody anti-CDK12 (human, clone Aq19.9.25). Stock concentration 1.45 mg/ml (1:5000 or 1:30000 for IHC; 1:1000 for IB; 1:2500 for IF);
- Homemade mouse monoclonal antibody anti-CDK12 (human and mouse, clone msBA54-73-11). Stock concentration 1.51 mg/ml (1:3000 or 1:30000 for IHC);
- anti-CK5 (ab53121) from Abcam, 1:2000 for IHC;
- anti-CK18 (sc-51582) from Santa Cruz Biotechnology, 1:100 for IHC;
- anti-p63 (DAK-p63) from Dako, 1:100 for IHC;
- anti-Ki67 (SP6)from Thermo Fisher Scientific,1:200 for IHC;
- anti-Giantin (Poly19243) from BioLegend, 1:500 for IF;
- anti-Vinculin (V9131) from Sigma, 1:10000 for IB;
- anti-PSAT1 (ABC950) from Sigma, 1:500 for IF;
- anti-MTHFD1 (HPA000704) from Sigma, 1:200 for IB.

Secondary Abs:
- Alexa Fluor 647 donkey anti rabbit IgG, Thermo Fisher, A-31573, 1:500 for IF.
- Cy3 donkey anti mouse IgG, Jackson ImmunoResearch, 715-165-150, 1:500 for IF.
- Anti-rabbit IgG HRP-linked, Cell Signaling, 7074, 1:2500 for IB.
- Anti-mouse IgG HRP-linked, Cell Signaling, 7076, 1:2500 for IB.

Validation

Primary Abs:
Homemade antibodies were validated in house through KD experiments validated with IB.
Validation statements available from manufacturers:
- anti-CK5: https://www.abcam.com/cytokeratin-5-antibody-cytoskeleton-marker-ab53121.html
- anti-CK18: https://www.scbt.com/p/cytokeratin-18-antibody-c-04
- anti-activated caspase-3: https://www.cellsignal.com/products/primary-antibodies/cleaved-caspase-3-asp175-antibody/9661
- anti-p63: https://www.agilent.com/en/product/immunohistochemistry/antibodies-controls/primary-antibodies/p63-protein-(dako-omnis)-76266
- anti-Ki67: https://www.thermofisher.com/antibody/product/Ki-67-Antibody-clone-SP6-Recombinant-Monoclonal/MA5-14520
- anti-Giantin: https://www.biolegend.com/en-us/products/anti-giantin-antibody-11064
- anti-Vinculin: https://www.sigmaaldrich.com/IT/en/product/sigma/v9131
- anti-PSAT1: https://www.merckmillipore.com/IT/it/product/Anti-PSAT1-Antibody,MM_NF-ABC950?ReferrerURL=https%3A%2F%2Fwww.google.com%2F&bd=1
- anti-MTHFD1: https://www.sigmaaldrich.com/IT/en/product/SIGMA/HPA000704

Secondary Abs:
- Alexa Fluor 647 donkey anti rabbit IgG: https://www.thermofisher.com/antibody/product/Donkey-anti-Rabbit-IgG-H-L-Highly-Cross-Adsorbed-Secondary-Antibody-Polyclonal/A-31573
- Cy3 donkey anti mouse IgG: https://www.jacksonimmuno.com/catalog/products/715-165-150
- Anti-IgG RABBIT HRP: https://www.cellsignal.com/products/secondary-antibodies/anti-rabbit-igg-hrp-linked-antibody/7074.
- Anti-IgG MOUSE HRP: https://www.cellsignal.com/products/secondary-antibodies/anti-mouse-igg-hrp-linked-antibody/7076

Eukaryotic cell lines

Policy information about cell lines

Cell line source(s)	MCF10A and BT474 cell lines were from the American Type Culture Collection (ATCC).
Authentication	All human cell lines were authenticated at each batch freezing by STR profiling (StemElite ID System, Promega)
Mycoplasma contamination	All cell lines were tested for mycoplasma by PCR (Uphoff and Drexler, 2002) and biochemical assay (MycoAlert, Lonza) and the results were negative.
Commonly misidentified lines (See ICLAC register)	NO

Animals and other organisms

Policy information about studies involving animals; ARRIVE guidelines recommended for reporting animal research

Laboratory animals	Mice strains used were: CDK12/Knock-in (KI) mice in FVB background; NOD/SCID IL2R gamma-chain null (NSG) mice. Patho-morphological analyses of CDK12-KI mammary glands were performed in female mice between 12 and 16 weeks old or in mice between 12 and 24 months old (Fig. 1 A,B). For xenograft experiments, NOD/SCID female mice were used between 16 and 24 weeks old. DMBA was administered to CDK12-KI and wild-type mice between 6 and 8 weeks old.
Wild animals	This study did not involve wild animals.
Field-collected samples	This study did not involve animals collected from the field.
Ethics oversight	All mouse experiments were conducted in a certified animal facility under the control of the institutional organism for animal welfare and ethical approach to animals in experimental procedures (Cogentech OBPA). Animal studies were conducted with the approval of Italian Ministry of Health and were performed in accordance with the Italian law (D.lgs. 26/2014), which enforces Dir. 2010/63/EU (Directive 2010/63/EU of the European Parliament and of the Council of 22 September 2010 on the protection of animals used for scientific purposes) and EU 86/609 directive.

Note that full information on the approval of the study protocol must also be provided in the manuscript.

Clinical data

Policy information about clinical studies

All manuscripts should comply with the ICMJEguidelines for publication of clinical research and a completedCONSORT checklist must be included with all submissions.

Clinical trial registration	IEO breast cancer patients from years 2000-2012 randomized to metronomic CM (cyclophosphamide+methotrexate) maintenance chemotherapy or no chemotherapy derive from an internationally registered clinical trial of the International Breast Cancer Study Group: IBCSG 22-00 (published in Colleoni M. et al., JCO 2016).
Study protocol	- A cohort of IEO breast cancer patients from years 2000-2012 enrolled in an internationally registered randomized clinical trial of the International Breast Cancer Study Group (IBCSG 22-00, Colleoni M. et al., JCO 2016) was used for the evaluation of CDK12 as a predictive biomarker of response to methotrexate-based therapy (described in Fig. 8c and Supplementary Fig. 5b). Clinical endpoint: cumulative incidence of distant metastasis.<br>- The cohort used for CDK12 immunohistochemistry and quantitative RT-PCR analysis is a retrospective consecutive collection of patients enrolled at IEO between years 1997-2000. Detailed description of the selection criteria and clinicopathological characteristics of this cohort have been previously described in Pece S. et al., EBioMedicine 2019. This cohort was used to assess the clinical value of CDK12 as a prognostic biomarker associated to different types of chemotherapy, as reported in Fig. 8b and Supplementary Fig. 4C-E and 5A). Clinical endpoint: cumulative incidence of distant metastasis.<br>- The three different neoadjuvant retrospective-consecutive cohorts of luminal, HER2-positive and triple-negative patients (described in Supplementary Fig. 5c) comprise IEO patients consecutively enrolled from years 2000-2016. These cohorts were used to investigate the association between CDK12 status and response to standard neoadjuvant treatments. Clinical endpoint: pathological complete response (PCR) vs. no-PCR (stable disease, disease progression and partial objective response).<br>- A retrospective consecutive cohorts of primary chemotherapy-resistant breast cancer patients longitudinally enrolled at IEO between years 1996-2015 was used to assess the efficacy of second-line CMF treatment according to CDK12 status (described in Fig. 8d and Supplementary Fig. 5d). Clinical endpoint: cumulative incidence of death.
Data collection	For IBCSG 22-00 patients, clinical and follow-up data were collected according to prespecified criteria, as described in Colleoni M. et al., JCO 2016).<br>For all the cohort studies, available clinical and pathological information included age, date at surgery, tumor characteristics (histological type, tumor size, nodal involvement, grade, perivascular infiltration, Ki67 proliferative index, estrogen-receptor and progesterone receptor status), and treatment modality (type of surgery, adjuvant radiotherapy, adjuvant endocrine therapy or chemotherapy, neoadjuvant chemotherapy). Patients were followed up with physical examination every 6 months, annual mammography and breast ultrasound, blood test every 6-12 months and further evaluation only in case of symptoms. When possible the status of women not presenting at the institute for scheduled follow-up visits for more that one year was obtained by telephone contact.
Outcomes	Cumulative incidence of Distant Metastasis was defined as the time from surgery to the appearance of distant metastasis or death from breast cancer as a first event. Second primary cancer or death from unknown causes or other causes were considered as

competing events. Cumulative incidence of Death was defined as the time from surgery to death. Pathological complete response (PCR) was defined as complete absence of residual infiltrating cancer cells or in situ carcinoma; no-PCR includes stable disease, disease progression on drug treatment and partial objective response.

The hazard ratios (HR) comparison were estimated with a Cox proportional hazards multivariable model adjusted for Grade (G1, G2 and G3), Ki-67 (Ki-67<14% and Ki-67≥14%), ERBB2 status (positive and negative), estrogen/progesterone receptor status [not expressed (Both 0) and expressed (ER>0 or PgR>0)], tumor size (pT1a/b, pT1c, pT2, pT3/pT4), number of positive lymph nodes (pN0, pN+) and age at surgery (<50 and ≥50). Analyses were carried out with the R (version 4.0.2) and JMP (version 14.0) software.
